# 3-(4-Fluoro­phenyl­sulfon­yl)-5-iodo-2,7-dimethyl-1-benzofuran

**DOI:** 10.1107/S1600536811052792

**Published:** 2011-12-10

**Authors:** Pil Ja Seo, Hong Dae Choi, Byeng Wha Son, Uk Lee

**Affiliations:** a Department of Chemistry, Dongeui University, San 24 Kaya-dong Busanjin-gu, Busan 614-714, Republic of Korea; b Department of Chemistry, Pukyong National University, 599-1 Daeyeon 3-dong, Nam-gu, Busan 608-737, Republic of Korea

## Abstract

In the title compound, C_16_H_12_FIO_3_S, the 4-fluoro­phenyl ring makes a dihedral angle of 72.31 (6)° with the mean plane of the benzofuran fragment. In the crystal, mol­ecules are linked by weak C—H⋯O hydrogen bonds, and by an I⋯I contact [3.7764 (3) Å]. The crystal structure also exhibits a weak C—I⋯π [3.901 (3) Å] inter­action and a slipped π–π inter­action between the furan and benzene rings of neighbouring mol­ecules [centroid–centroid distance = 3.845 (3), inter­planar distance = 3.555 (3) and slippage = 1.465 (3) Å].

## Related literature

For the biological activity of benzofuran compounds, see: Aslam *et al.* (2009 ▶); Galal *et al.* (2009 ▶); Khan *et al.* (2005 ▶). For natural products with benzofuran rings, see: Akgul & Anil (2003 ▶); Soekamto *et al.* (2003 ▶). For related structures, see: Choi *et al.* (2008 ▶, 2011 ▶).
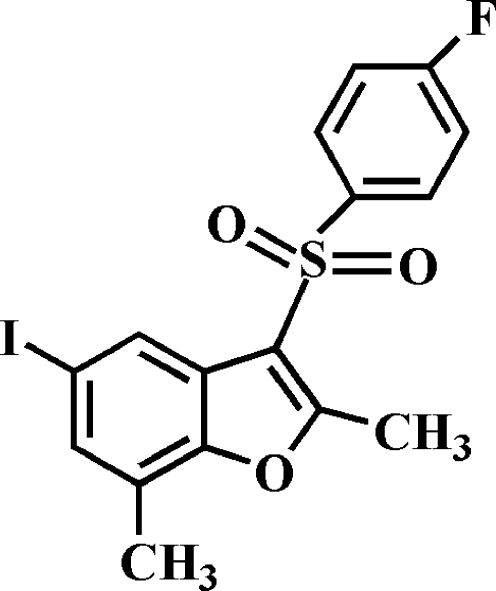


## Experimental

### 

#### Crystal data

C_16_H_12_FIO_3_S
*M* *_r_* = 430.22
Triclinic, 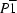

*a* = 7.5839 (2) Å
*b* = 10.2443 (3) Å
*c* = 10.4651 (3) Å
α = 79.424 (1)°
β = 75.652 (1)°
γ = 80.361 (1)°
*V* = 767.94 (4) Å^3^
*Z* = 2
Mo *K*α radiation
μ = 2.24 mm^−1^
*T* = 173 K
0.32 × 0.30 × 0.18 mm

#### Data collection

Bruker SMART APEXII CCD diffractometer
Absorption correction: multi-scan (*SADABS*; Bruker, 2009 ▶) *T*_min_ = 0.534, *T*_max_ = 0.689
13962 measured reflections
3785 independent reflections
3585 reflections with *I* > 2σ(*I*)
*R*_int_ = 0.028

#### Refinement

*R*[*F* ^2^ > 2σ(*F* ^2^)] = 0.022
*wR*(*F* ^2^) = 0.057
*S* = 1.08
3785 reflections
201 parameters
H-atom parameters constrained
Δρ_max_ = 0.47 e Å^−3^
Δρ_min_ = −0.72 e Å^−3^

### 

Data collection: *APEX2* (Bruker, 2009 ▶); cell refinement: *SAINT* (Bruker, 2009 ▶); data reduction: *SAINT*; program(s) used to solve structure: *SHELXS97* (Sheldrick, 2008 ▶); program(s) used to refine structure: *SHELXL97* (Sheldrick, 2008 ▶); molecular graphics: *ORTEP-3* (Farrugia, 1997 ▶) and *DIAMOND* (Brandenburg, 1998 ▶); software used to prepare material for publication: *SHELXL97*.

## Supplementary Material

Crystal structure: contains datablock(s) global, I. DOI: 10.1107/S1600536811052792/bh2405sup1.cif

Structure factors: contains datablock(s) I. DOI: 10.1107/S1600536811052792/bh2405Isup2.hkl

Supplementary material file. DOI: 10.1107/S1600536811052792/bh2405Isup3.cml

Additional supplementary materials:  crystallographic information; 3D view; checkCIF report

## Figures and Tables

**Table 1:** Hydrogen-bond geometry (Å, °)

*D*—H⋯*A*	*D*—H	H⋯*A*	*D*⋯*A*	*D*—H⋯*A*
C12—H12⋯O3^i^	0.95	2.54	3.253 (2)	132
C15—H15⋯O2^ii^	0.95	2.52	3.294 (3)	139

Symmetry codes: (i) 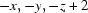; (ii) 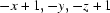.

**Fig. 1.**
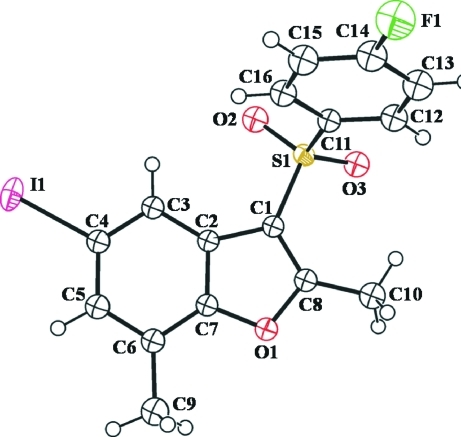
The molecular structure of the title compound with displacement ellipsoids at the 50% probability level. H atoms are presented as small spheres of arbitrary radius.

**Fig. 2.**
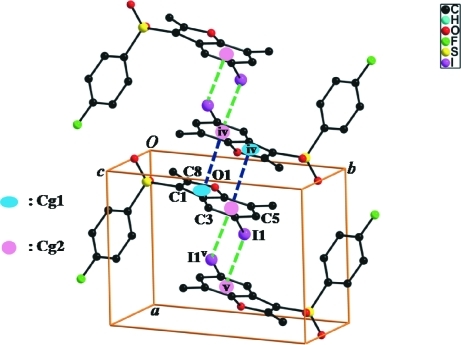
A view of the C—H···O and I···I interactions (dotted lines) in the crystal structure of the title compound. H atoms non-participating in hydrogen-bonding were omitted for clarity [Symmetry codes: (i) -x+1, -y+1, -z; (ii) -x, -y, -z+2; (iii) -x+1, -y, -z+1].

**Fig. 3.**
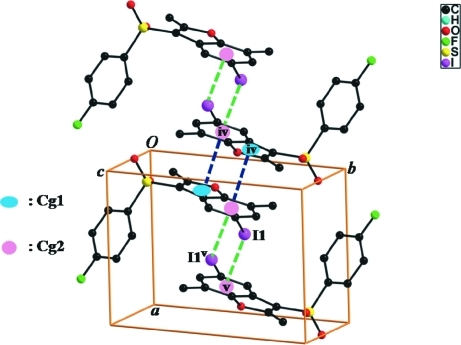
A view of the C—I···π and π–π interactions (dotted lines) in the crystal structure of the title compound. H atoms were omitted for clarity [Symmetry codes: (iv) -x, -y+1, -z+1; (v) -x+1, -y+1, -z+1].

## supplementary crystallographic information

### Comment

Recently, benzofuran derivatives have drawn much attention due to their valuable pharmacological properties such as antibacterial and antifungal, antitumor and antiviral, and antimicrobial activities (Aslam *et al.*, 2009; Galal *et al.*, 2009; Khan *et al.*, 2005). These benzofuran derivatives occur in a wide range of natural products (Akgul & Anil, 2003; Soekamto *et al.*, 2003). As a part of our ongoing study of benzofuran derivatives containing either 3-phenylsulfonyl (Choi *et al.*, 2008) or 3-(4-fluorophenylsulfinyl) (Choi *et al.*, 2011) substituents, we report herein the crystal structure of the title compound.

In the title molecule (Fig. 1), the benzofuran unit is essentially planar, with a mean deviation of 0.007 (2) Å from the least-squares plane defined by the nine constituent atoms. The dihedral angle formed by the 4-fluorophenyl ring and the mean plane of the benzofurn fragment is 72.31 (6)°. The crystal packing (Fig. 2) is stabilized by weak intermolecular C—H···O hydrogen bonds (see Table 1), and by an I···I contact at 3.7764 (3) Å. The crystal packing (Fig. 3) is further stabilized by a weak C—I···π interaction between the iodine and the benzene ring of an adjacent molecule, with a C4—I1···Cg2^v^ [3.901 (3) Å] (Cg2 is the centroid of the C2···C7 benzene ring). The crystal packing (Fig. 3) is also exhibits a weak slipped π–π interaction between the furan and benzene rings of adjacent molecules, with a Cg1···Cg2^iv^ distance of 3.845 (3) Å and an interplanar distance of 3.555 (3) Å resulting in a slippage of 1.465 (3) Å (Cg1 is the centroid of the C1/C2/C7/O1/C8 furan ring).

### Experimental

77% 3-chloroperoxybenzoic acid (381 mg, 1.7 mmol) was added in small portions to a stirred solution of 3-(4-fluorophenylsulfanyl)-5-iodo-2,7-dimethyl-1-benzofuran (318 mg, 0.8 mmol) in dichloromethane (30 mL) at 273 K. After being stirred at room temperature for 8 h, the mixture was washed with saturated sodium bicarbonate solution and the organic layer was separated, dried over magnesium sulfate, filtered and concentrated at reduced pressure. The residue was purified by column chromatography (hexane–ethyl acetate, 4:1 v/v) to afford the title compound as a colorless solid [yield 71%, m.p. 430–431 K; *R*_f_ = 0.53 (hexane–ethyl acetate, 4:1 v/v)]. Single crystals suitable for X-ray diffraction were prepared by slow evaporation of a solution of the title compound in ethyl acetate at room temperature.

### Refinement

All H atoms were positioned geometrically and refined using a riding model, with C—H = 0.95 Å fo the aryl and 0.98 Å for the methylene H atoms. *U*_iso_(H) =1.2*U*_eq_(C) for the aryl and 1.5*U*_eq_(C) for the methyl H atoms.

### Crystal data

**Table:**

C_16_H_12_FIO_3_S	*Z* = 2
*M**_r_* = 430.22	*F*(000) = 420
Triclinic, *P*1	*D*_x_ = 1.861 Mg m^−^^3^
Hall symbol: -P 1	Melting point: 430 K
*a* = 7.5839 (2) Å	Mo *K*α radiation, λ = 0.71073 Å
*b* = 10.2443 (3) Å	Cell parameters from 9929 reflections
*c* = 10.4651 (3) Å	θ = 2.7–28.3°
α = 79.424 (1)°	µ = 2.24 mm^−^^1^
β = 75.652 (1)°	*T* = 173 K
γ = 80.361 (1)°	Block, colourless
*V* = 767.94 (4) Å^3^	0.32 × 0.30 × 0.18 mm

### Data collection

**Table:**

Bruker SMART APEXII CCD diffractometer	3785 independent reflections
Radiation source: rotating anode	3585 reflections with *I* > 2σ(*I*)
graphite multilayer	*R*_int_ = 0.028
Detector resolution: 10.0 pixels mm^-1^	θ_max_ = 28.3°, θ_min_ = 2.0°
φ and ω scans	*h* = −9→10
Absorption correction: multi-scan (*SADABS*; Bruker, 2009)	*k* = −13→13
*T*_min_ = 0.534, *T*_max_ = 0.689	*l* = −13→13
13962 measured reflections	

### Refinement

**Table:**

Refinement on *F*^2^	Primary atom site location: structure-invariant direct methods
Least-squares matrix: full	Secondary atom site location: difference Fourier map
*R*[*F*^2^ > 2σ(*F*^2^)] = 0.022	Hydrogen site location: difference Fourier map
*wR*(*F*^2^) = 0.057	H-atom parameters constrained
*S* = 1.08	*w* = 1/[σ^2^(*F*_o_^2^) + (0.028*P*)^2^ + 0.3904*P*] where *P* = (*F*_o_^2^ + 2*F*_c_^2^)/3
3785 reflections	(Δ/σ)_max_ = 0.001
201 parameters	Δρ_max_ = 0.47 e Å^−^^3^
0 restraints	Δρ_min_ = −0.72 e Å^−^^3^
0 constraints	

### Fractional atomic coordinates and isotropic or equivalent isotropic displacement parameters (Å^2^)

**Table:**

	*x*	*y*	*z*	*U*_iso_*/*U*_eq_	
I1	0.458085 (17)	0.514608 (14)	0.182660 (11)	0.03682 (6)	
S1	0.09268 (6)	0.13784 (4)	0.70600 (4)	0.02423 (9)	
F1	0.7392 (2)	−0.17671 (16)	0.86383 (18)	0.0602 (4)	
O1	0.07637 (18)	0.50285 (12)	0.77645 (12)	0.0243 (2)	
O2	0.1076 (2)	0.12200 (14)	0.56993 (14)	0.0337 (3)	
O3	−0.06489 (19)	0.09921 (14)	0.80627 (15)	0.0332 (3)	
C1	0.1071 (2)	0.30519 (17)	0.70547 (17)	0.0227 (3)	
C2	0.1812 (2)	0.39985 (16)	0.59204 (17)	0.0219 (3)	
C3	0.2651 (2)	0.39515 (18)	0.45767 (17)	0.0242 (3)	
H3	0.2828	0.3154	0.4195	0.029*	
C4	0.3210 (2)	0.51397 (19)	0.38343 (17)	0.0259 (3)	
C5	0.2946 (3)	0.63304 (18)	0.43641 (19)	0.0281 (4)	
H5	0.3345	0.7114	0.3802	0.034*	
C6	0.2110 (3)	0.63949 (17)	0.56963 (19)	0.0261 (3)	
C7	0.1583 (2)	0.51938 (17)	0.64268 (17)	0.0226 (3)	
C8	0.0489 (2)	0.37130 (17)	0.81325 (18)	0.0242 (3)	
C9	0.1834 (3)	0.76435 (19)	0.6319 (2)	0.0358 (4)	
H9A	0.2786	0.7597	0.6817	0.054*	
H9B	0.0623	0.7723	0.6928	0.054*	
H9C	0.1914	0.8425	0.5620	0.054*	
C10	−0.0272 (3)	0.3303 (2)	0.95636 (18)	0.0326 (4)	
H10A	−0.0523	0.2373	0.9712	0.049*	
H10B	−0.1414	0.3885	0.9853	0.049*	
H10C	0.0616	0.3379	1.0077	0.049*	
C11	0.2897 (2)	0.04748 (16)	0.75598 (17)	0.0239 (3)	
C12	0.2733 (3)	−0.02336 (19)	0.88349 (19)	0.0318 (4)	
H12	0.1580	−0.0191	0.9453	0.038*	
C13	0.4256 (3)	−0.1004 (2)	0.9203 (2)	0.0391 (5)	
H13	0.4169	−0.1514	1.0067	0.047*	
C14	0.5908 (3)	−0.1011 (2)	0.8280 (2)	0.0380 (4)	
C15	0.6120 (3)	−0.0298 (2)	0.7025 (2)	0.0368 (4)	
H15	0.7288	−0.0318	0.6425	0.044*	
C16	0.4587 (3)	0.0453 (2)	0.66519 (19)	0.0304 (4)	
H16	0.4686	0.0951	0.5783	0.037*	

### Atomic displacement parameters (Å^2^)

**Table:**

	*U*^11^	*U*^22^	*U*^33^	*U*^12^	*U*^13^	*U*^23^
I1	0.03090 (8)	0.05323 (10)	0.02085 (7)	−0.00336 (6)	−0.00149 (5)	0.00069 (5)
S1	0.0273 (2)	0.02141 (18)	0.0241 (2)	−0.00622 (16)	−0.00472 (16)	−0.00224 (15)
F1	0.0409 (8)	0.0588 (9)	0.0736 (11)	0.0116 (7)	−0.0215 (7)	0.0040 (8)
O1	0.0276 (6)	0.0228 (6)	0.0215 (6)	−0.0025 (5)	−0.0034 (5)	−0.0042 (4)
O2	0.0468 (8)	0.0291 (7)	0.0292 (7)	−0.0055 (6)	−0.0134 (6)	−0.0071 (5)
O3	0.0276 (7)	0.0322 (7)	0.0373 (8)	−0.0115 (5)	−0.0017 (6)	0.0002 (6)
C1	0.0243 (8)	0.0213 (7)	0.0219 (8)	−0.0038 (6)	−0.0043 (6)	−0.0018 (6)
C2	0.0204 (8)	0.0220 (7)	0.0225 (8)	−0.0020 (6)	−0.0046 (6)	−0.0024 (6)
C3	0.0237 (8)	0.0260 (8)	0.0221 (8)	−0.0020 (6)	−0.0043 (6)	−0.0032 (6)
C4	0.0227 (8)	0.0328 (9)	0.0199 (8)	−0.0028 (7)	−0.0035 (6)	−0.0002 (7)
C5	0.0273 (9)	0.0271 (8)	0.0282 (9)	−0.0061 (7)	−0.0073 (7)	0.0043 (7)
C6	0.0264 (9)	0.0223 (8)	0.0299 (9)	−0.0037 (6)	−0.0080 (7)	−0.0016 (7)
C7	0.0215 (8)	0.0235 (8)	0.0220 (8)	−0.0017 (6)	−0.0040 (6)	−0.0033 (6)
C8	0.0238 (8)	0.0247 (8)	0.0239 (8)	−0.0036 (6)	−0.0054 (6)	−0.0026 (6)
C9	0.0468 (12)	0.0237 (8)	0.0384 (11)	−0.0065 (8)	−0.0107 (9)	−0.0050 (8)
C10	0.0386 (11)	0.0356 (10)	0.0208 (8)	−0.0062 (8)	−0.0015 (7)	−0.0028 (7)
C11	0.0271 (9)	0.0191 (7)	0.0240 (8)	−0.0036 (6)	−0.0033 (6)	−0.0026 (6)
C12	0.0320 (10)	0.0310 (9)	0.0272 (9)	−0.0056 (7)	−0.0011 (7)	0.0026 (7)
C13	0.0424 (12)	0.0353 (10)	0.0342 (10)	−0.0031 (9)	−0.0099 (9)	0.0088 (8)
C14	0.0342 (11)	0.0324 (10)	0.0470 (12)	0.0029 (8)	−0.0133 (9)	−0.0057 (9)
C15	0.0282 (10)	0.0413 (11)	0.0382 (11)	−0.0025 (8)	−0.0010 (8)	−0.0100 (9)
C16	0.0308 (10)	0.0329 (9)	0.0252 (8)	−0.0066 (7)	−0.0006 (7)	−0.0037 (7)

### Geometric parameters (Å, °)

**Table:**

I1—C4	2.1000 (17)	C6—C9	1.501 (3)
I1—I1^i^	3.7764 (3)	C8—C10	1.475 (2)
S1—O2	1.4372 (14)	C9—H9A	0.9800
S1—O3	1.4388 (14)	C9—H9B	0.9800
S1—C1	1.7349 (17)	C9—H9C	0.9800
S1—C11	1.7633 (19)	C10—H10A	0.9800
F1—C14	1.348 (3)	C10—H10B	0.9800
O1—C8	1.368 (2)	C10—H10C	0.9800
O1—C7	1.375 (2)	C11—C12	1.384 (3)
C1—C8	1.364 (2)	C11—C16	1.393 (2)
C1—C2	1.447 (2)	C12—C13	1.382 (3)
C2—C7	1.390 (2)	C12—H12	0.9500
C2—C3	1.398 (2)	C13—C14	1.379 (3)
C3—C4	1.388 (2)	C13—H13	0.9500
C3—H3	0.9500	C14—C15	1.367 (3)
C4—C5	1.395 (3)	C15—C16	1.382 (3)
C5—C6	1.390 (3)	C15—H15	0.9500
C5—H5	0.9500	C16—H16	0.9500
C6—C7	1.388 (2)		
			
C4—I1—I1^i^	159.65 (5)	O1—C8—C10	115.71 (15)
O2—S1—O3	119.47 (9)	C6—C9—H9A	109.5
O2—S1—C1	106.66 (8)	C6—C9—H9B	109.5
O3—S1—C1	108.80 (8)	H9A—C9—H9B	109.5
O2—S1—C11	107.68 (9)	C6—C9—H9C	109.5
O3—S1—C11	107.59 (9)	H9A—C9—H9C	109.5
C1—S1—C11	105.89 (8)	H9B—C9—H9C	109.5
C8—O1—C7	107.10 (13)	C8—C10—H10A	109.5
C8—C1—C2	107.64 (15)	C8—C10—H10B	109.5
C8—C1—S1	125.62 (13)	H10A—C10—H10B	109.5
C2—C1—S1	126.74 (13)	C8—C10—H10C	109.5
C7—C2—C3	119.58 (16)	H10A—C10—H10C	109.5
C7—C2—C1	104.40 (14)	H10B—C10—H10C	109.5
C3—C2—C1	136.01 (16)	C12—C11—C16	120.97 (18)
C4—C3—C2	116.04 (16)	C12—C11—S1	119.54 (14)
C4—C3—H3	122.0	C16—C11—S1	119.47 (14)
C2—C3—H3	122.0	C13—C12—C11	119.60 (18)
C3—C4—C5	123.23 (16)	C13—C12—H12	120.2
C3—C4—I1	118.75 (13)	C11—C12—H12	120.2
C5—C4—I1	118.01 (13)	C14—C13—C12	118.06 (19)
C6—C5—C4	121.44 (16)	C14—C13—H13	121.0
C6—C5—H5	119.3	C12—C13—H13	121.0
C4—C5—H5	119.3	F1—C14—C15	118.3 (2)
C7—C6—C5	114.51 (16)	F1—C14—C13	118.0 (2)
C7—C6—C9	122.07 (17)	C15—C14—C13	123.6 (2)
C5—C6—C9	123.40 (17)	C14—C15—C16	118.16 (19)
O1—C7—C6	124.09 (15)	C14—C15—H15	120.9
O1—C7—C2	110.72 (14)	C16—C15—H15	120.9
C6—C7—C2	125.19 (16)	C15—C16—C11	119.55 (19)
C1—C8—O1	110.13 (15)	C15—C16—H16	120.2
C1—C8—C10	134.10 (16)	C11—C16—H16	120.2
			
O2—S1—C1—C8	160.36 (16)	C3—C2—C7—O1	−178.98 (15)
O3—S1—C1—C8	30.24 (19)	C1—C2—C7—O1	−0.15 (19)
C11—S1—C1—C8	−85.14 (18)	C3—C2—C7—C6	0.7 (3)
O2—S1—C1—C2	−19.34 (19)	C1—C2—C7—C6	179.55 (17)
O3—S1—C1—C2	−149.46 (16)	C2—C1—C8—O1	1.5 (2)
C11—S1—C1—C2	95.16 (17)	S1—C1—C8—O1	−178.27 (13)
C8—C1—C2—C7	−0.8 (2)	C2—C1—C8—C10	−175.6 (2)
S1—C1—C2—C7	178.95 (14)	S1—C1—C8—C10	4.7 (3)
C8—C1—C2—C3	177.7 (2)	C7—O1—C8—C1	−1.56 (19)
S1—C1—C2—C3	−2.5 (3)	C7—O1—C8—C10	176.10 (16)
C7—C2—C3—C4	0.2 (3)	O2—S1—C11—C12	−140.80 (15)
C1—C2—C3—C4	−178.18 (19)	O3—S1—C11—C12	−10.81 (17)
C2—C3—C4—C5	−0.9 (3)	C1—S1—C11—C12	105.39 (16)
C2—C3—C4—I1	177.53 (12)	O2—S1—C11—C16	37.34 (17)
I1^i^—I1—C4—C3	63.4 (2)	O3—S1—C11—C16	167.33 (14)
I1^i^—I1—C4—C5	−118.06 (16)	C1—S1—C11—C16	−76.46 (16)
C3—C4—C5—C6	0.8 (3)	C16—C11—C12—C13	−1.8 (3)
I1—C4—C5—C6	−177.66 (14)	S1—C11—C12—C13	176.32 (16)
C4—C5—C6—C7	0.1 (3)	C11—C12—C13—C14	1.4 (3)
C4—C5—C6—C9	178.53 (19)	C12—C13—C14—F1	−179.7 (2)
C8—O1—C7—C6	−178.67 (17)	C12—C13—C14—C15	0.1 (4)
C8—O1—C7—C2	1.04 (19)	F1—C14—C15—C16	178.6 (2)
C5—C6—C7—O1	178.82 (16)	C13—C14—C15—C16	−1.2 (3)
C9—C6—C7—O1	0.4 (3)	C14—C15—C16—C11	0.8 (3)
C5—C6—C7—C2	−0.8 (3)	C12—C11—C16—C15	0.7 (3)
C9—C6—C7—C2	−179.31 (18)	S1—C11—C16—C15	−177.42 (15)

Symmetry codes: (i) −*x*+1, −*y*+1, −*z*.

### Hydrogen-bond geometry (Å, °)

**Table:**

*D*—H···*A*	*D*—H	H···*A*	*D*···*A*	*D*—H···*A*
C12—H12···O3^ii^	0.95	2.54	3.253 (2)	132.
C15—H15···O2^iii^	0.95	2.52	3.294 (3)	139.

Symmetry codes: (ii) −*x*, −*y*, −*z*+2; (iii) −*x*+1, −*y*, −*z*+1.
